# Cleavage of Oligonucleotides Containing a P3’→N5’ Phosphoramidate Linkage Mediated by Single-Stranded Oligonucleotide Templates

**DOI:** 10.3390/molecules161210695

**Published:** 2011-12-20

**Authors:** Kosuke Ramon Ito, Tetsuya Kodama, Futaba Makimura, Noritsugu Hosoki, Tomohisa Osaki, Ayako Orita, Takeshi Imanishi, Satoshi Obika

**Affiliations:** 1 Graduate School of Pharmaceutical Sciences, Osaka University, 1-6 Yamadaoka, Suita, Osaka 565-0871, Japan; 2 BNA Inc., 7-7-20 Saito-asagi, Ibaraki, Osaka 567-0085, Japan

**Keywords:** bridged nucleic acids, DNA sensing, P3’→N5’ phosphoramidate

## Abstract

Double-stranded DNA (dsDNA) templates can hybridize to and accelerate cleavage of oligonucleotides containing a P3’→N5’ phosphoramidate (P-N) linkage. This dsDNA-templated cleavage of P-N linkages could be due to conformational strain placed on the linkage upon triplex formation. To determine whether duplex formation also induced conformational strain, we examined the reactivity of the oligonucleotides with a P-N linkage in the presence of single-stranded templates, and compared these reactions to those with dsDNA templates. P-N oligonucleotides that are cleaved upon duplex formation could be used as probes to detect single-stranded nucleic acids.

## 1. Introduction

Detection of nucleic acids via nucleic acid-templated reactions has attracted substantial interest [1,2,3,4,5,6,7,8,9,10,11,12,13,14,15,16,17,18,19,20,21,22,23,24]. Most of these techniques employ ligation of probes [1,2,3,4,5,6,7,8,9,10,11] and subsequent transfer [12,13,14], release [15,16,17], or activation [18,19,20,21,22,23,24] of a reporter group upon hybridization of the probes to the template DNA and/or RNA, based on an effective-molecularity approach [25]. When reactants are placed in close proximity to template nucleic acids via sequence-specific hybridization, the effective molecularity of the reactants increases.

**Figure 1:**
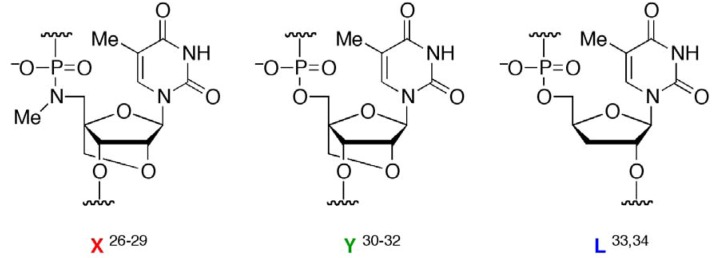
Structures of 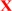 (5’-amino-2’,4’-BNA), 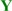 (2’,4’-BNA/LNA), and 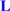 (2’,5’-linked DNA).

**Figure 2:**
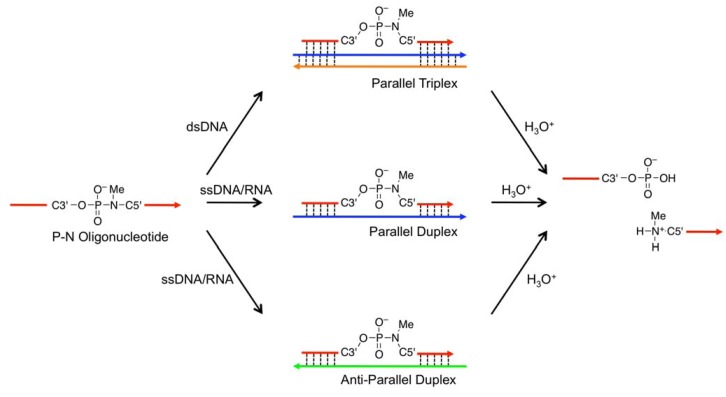
Schematic representation of nucleic acid-templated hydrolysis of phosphoramidate. Template nucleic acids can be dsDNA or parallel or anti-parallel single-stranded nucleic acids.

We presented a new type of DNA-templated reaction-based DNA detection; this method involves probe cleavage that is templated by double-stranded DNA (dsDNA) [26,27,28,29]. In this method, cleavage reactions are accelerated because of conformational strain induced by triplex formation, not because of the effective molecularity. We utilized triplex-forming oligonucleotides (TFOs) containing 5’-amino-2’,4’-BNA (Figure 1, 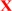) as probes; the TFOs have a P3’→N5’ phosphoramidate (P-N) linkage in the backbone. This linkage was more susceptible to acid-mediated hydrolysis upon triplex formation, and the enhanced susceptibility was due to conformational strain on the P-N linkage induced by triplex formation. Previously, we examined the effects of chemical modifications that alter the microenvironment around the P-N linkage and change the extent of the conformational strain; these chemical modifications had substantial effects on the observed pseudo first-order rate constants (*k*_obs_s) of the hydrolysis with the dsDNA templates [29]. These findings indicated that when the P-N linkage is subjected to sufficient strain, the linkage promptly breaks upon hybridization to the template. We hypothesized that duplex formation, like triplex formation, could induce conformational strain when oligonucleotides have a certain chemical modification and that such oligonucleotides may be selectively cleaved in the presence of single-stranded templates and, therefore, may be used as probes to detect single-stranded nucleic acids (Figure 2).

Here, we prepared several oligonucleotides containing a moiety (designated 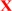, Figure 1) in the middle of a sequence with one of two chemical modifications, 2’,4’-BNA/LNA [30,31,32] (designated 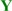) or 2’,5’-linked DNA [33,34] (designated 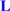), on adjacent residues (Table 1). The reactivity of these oligonucleotides in the presence of single-stranded DNA (ssDNA) or ssRNA templates was compared with their reactivity in the presence of parallel double-stranded DNA (**PDD**) templates and in the absence of any template. The parallel (Hoogsteen motif) single-stranded DNA and RNA (**PSD** and **PSR**, respectively) and anti-parallel (Watson-Crick motif) single-stranded DNA and RNA (**ASD** and **ASR**) were prepared as templates (see Table 1 caption). The formation of different motifs of duplexes was expected to have different effects on the reactivity of the hydrolysis depending on the extent of the strain.

## 2. Results

### 2.1. UV Melting Experiments

**Figure 3:**
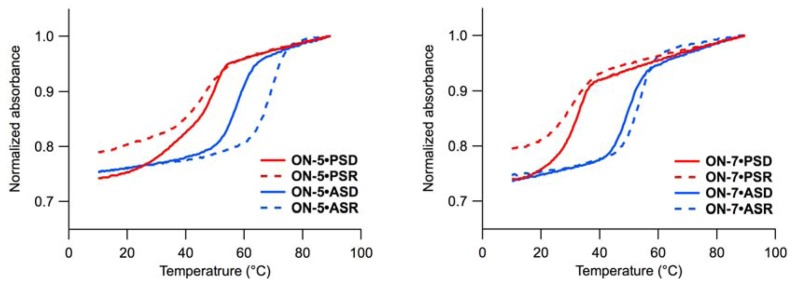
Melting profiles of **ON-5** (left) and **ON-7** (right) with **PSD** (red, solid), **PSR** (red, dashed), **ASD** (blue, solid), and **ASR** (blue, dashed). Conditions: 140 mM KCl, 10 mM MgCl_2_, 1.0 mM sodium phosphate, 10 mM sodium citrate buffer, 1.5 µM each strand, pH 6.0.

Initially, we evaluated the ability of **ON-1**–**ON-8** to form duplexes with oligonucleotide templates. Although we tried to assess the affinity of **ON-1**–**ON-8** for template oligonucleotides under the acidic conditions in which the cleavage reactions were performed, it was not possible due to acid lability of **ON-1**–**ON-8** [29]. Therefore, we performed the melting experiments using **ON-0**, which has no P-N linkage, at pH 4.0 and under milder conditions (pH 6.0). The *T*_m_s of **ON-0** at pH 6.0 and at pH 4.0 were compared with those of at pH 6.0 to estimate the stability of **ON-1**–**ON-8**-containing duplexes at pH 4.0. The *T*_m_ of each oligonucleotide is presented in Table 1, and the melting profiles are given in Figure 3 and Figure S1.

The comparison between the heating and cooling processes provided information on hysteresis. Under slightly acidic conditions (pH 6.0), association and dissociation of **ON-0**–**ON-8** with parallel single-stranded RNA (**PSR**), anti-parallel single-stranded DNA (**ASD**) and RNA (**ASR**) were reversible, and no significant hysteresis was observed. In contrast, **ON-0**–**ON-8** showed hysteresis in the melting experiments with parallel single-stranded DNA (**PSD**). At pH 4.0, hysteresis was observed for duplexes **ON-0**•**PSD** and **ON-0**•**ASD**.

The effects of acidic conditions on the stability of the duplexes varied depending on the template. The duplex **ON-0**•**PSD** was stabilized at pH 4.0 (+15 °C), while the duplex **ON-0**•**PSR** was not stable under acidic conditions (*T*_m_ not determined). Anti-parallel duplexes were destabilized under acidic conditions, and the extent of the destabilization was much larger for the duplex **ON-0**•**ASR** (−18 °C) than that for **ON-0**•**ASD** (−4 °C).

**Table 1:** *T*_m_s of duplexes containing an **ON-0–ON-8** oligonucleotide and **ASD**, **ASR**, **PSD**, or **PSR** ^a^.

ON	Sequence (5’ to 3’) ^b^	*T*_m_ (Δ *T*_m_) in °C with			
		PSD ^c^	PSR ^c^	ASD ^c^	ASR ^c^
**ON-0**	TTTTT^m^CTTT^m^CT^m^CT^m^CT	33 (-)	34 (-)	52 (-)	54 (-)
**ON-0 ^d^**	TTTTT^m^CTTT^m^CT^m^CT^m^CT	48 (+15)	n.d. ^e^	48 (−4)	36 (−18)
**ON-1**	TTTTT^m^CT 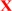 T^m^CT^m^CT^m^CT	38 (+5)	34 (±0)	54 (+2)	57 (+3)
**ON-2**	TTTTT^m^C 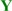 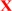 T^m^CT^m^CT^m^CT	37 (+4)	37 (+3)	53 (+1)	60 (+6)
**ON-3**	TTTTT^m^CT 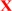 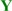 ^m^CT^m^CT^m^CT	41 (+8)	40 (+6)	55 (+3)	62 (+8)
**ON-4**	TTTTT^m^C 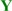 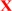 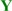 ^m^CT^m^CT^m^CT	44 (+11)	44 (+10)	54 (+2)	65 (+11)
**ON-5**	TTTT 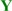 ^m^CT 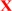 T^m^C 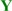 ^m^CT^m^CT	45 (+12)	46 (+12)	57 (+5)	69 (+15)
**ON-6**	TTTTT^m^C 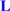 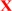 T^m^CT^m^CT^m^CT	33 (±0)	34 (±0)	47 (−5)	55 (+1)
**ON-7**	TTTTT^m^CT 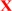 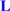 ^m^CT^m^CT^m^CT	31 (−2)	30 (−4)	49 (−3)	53 (−1)
**ON-8**	TTTTT^m^C 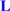 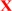 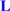 ^m^CT^m^CT^m^CT	31 (−2)	29 (−5)	43 (−9)	51 (−3)

^a^ Conditions: 140 mM KCl, 10 mM MgCl_2_, 1.0 mM sodium phosphate, 10 mM sodium citrate, 1.5 µM each strand, pH 6.0; ^b^ 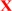, 5’-amino-2’,4’-BNA (NMe); 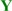, 2’,4’-BNA/LNA; 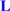, 2’,5’-linked DNA; ^m^C, 5-MedC; ^c^ **PSD** (parallel single-stranded DNA), 5’-d(AAAAAGAAAGAGAGA)-3’; **PSR** (parallel single-stranded RNA), 5’-r(AAAAAGAAAGAGAGA)-3’; **ASD** (anti-parallel single-stranded DNA), 5’-d(AGAGAGAAAGAAAAA)-3’; **ASR** (anti-parallel single-stranded RNA), 5’-r(AGAGAGAAAGAAAAA)-3’; ^d^ *T*_m_ measured at pH 4.0, for detail see experimental section; e: Not determined due to low stability (*T*_m_ < 25 °C).

As we expected, introduction of 2’,4’-BNA/LNA (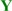) (**ON-2**–**ON-5**) stabilized the duplexes in most cases [30,31,32], and the stabilizing effects were more apparent for the duplexes with **PSD**, **PSR**, and **ASR** than those with **ASD**. Comparisons between **ON-2** and **ON-3** *T*_m_s revealed that a 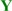 positioned just 3’ of 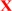 (5’-amino-2’,4’-BNA) stabilized all duplexes to a larger extent than a 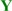 positioned just 5’ of 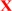. Insertion of two residues between 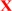 and 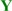 had greater stabilizing effects in all types of duplexes tested here (**ON-4** *vs.* **ON-5**). Comparison of *T*_m_s of duplexes containing 2’,5’-linked DNA (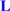) with those of **ON-1** revealed that introduction of 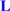 is destabilizing in most cases. The destabilization was less pronounced for the duplexes with **ASR**, as reported previously [35,36,37]. The melting curves of the duplexes consisting of **PSD** and **ON-3**, **ON-4**, or **ON-8** showed some two-transition character.

### 2.2. Hydrolysis Experiments

**Figure 4:**
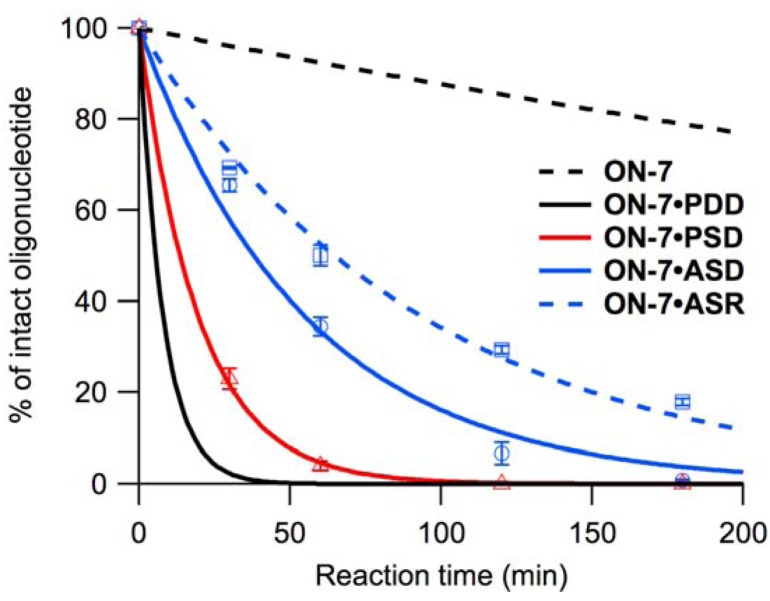
Cleavage profiles of **ON-7**, with no template (black, dashed), **PDD** (black, solid), **PSD** (red, solid), **ASD** (blue, solid), and **ASR** (blue, dashed) at pH 4.0, 40 °C. Black lines are drawn using the kinetic parameters given in Table 2.

We performed hydrolysis experiments with **ON-1**–**ON-8** and single-stranded oligonucleotide templates (**PSD**, **ASD** and **ASR**) under conditions identical to those described previously for double-stranded DNA-templated reactions (pH 4.0, 40 °C), [28,29]. However, based on the duplex-forming ability of the oligonucleotides estimated from the UV melting experiments, the duplexes containing **ASR** were estimated to be destabilized under acidic conditions. Additionally, duplexes containing **PSD** did not exhibit sufficiently high thermal stability at pH 6.0, although they were estimated to be much more stable under acidic conditions. Therefore, the hydrolysis experiments using an **ON-1**–**ON-8** oligonucleotide and **ASR** or **PSD** were performed at 20 °C, because the duplexes were assumed to be sufficiently stable at this temperature. The duplexes with **PSR** were estimated to be significantly unstable under acidic conditions, and the reactivity on **PSR** was not evaluated. The *k*_obs_s of each oligonucleotide at 40 °C and 20 °C are shown in Table 2 and Table 3, respectively, and the cleavage profiles are shown in Figure 4, Figure S2, S3 and S4.

**Table 2:** Observed pseudo first-order rate constants of each oligonucleotide at pH 4.0, 40 °C ^a^.

ON	*k*_obs_ × 10^3^ (s^−1^) in the presence of				
	No template ^b^	PDD ^b,c^	PSD	ASD	ASR
**ON-1**	0.027 ± 0.005	0.77 ± 0.03	0.57 ± 0.09	0.14 ± 0.01	0.17 ± <0.01
**ON-2**	0.017 ± 0.006	0.051 ± 0.013	0.025 ± 0.001	0.020 ± 0.002	0.032 ± 0.001
**ON-3**	0.038 ± 0.012	1.4 ± 0.4	0.60 ± 0.05	0.10 ± <0.01	0.19 ± <0.01
**ON-4**	0.026 ± 0.003	0.044 ± 0.025	0.032 ± 0.003	0.015 ± 0.003	n.d. ^d^
**ON-5**	0.029 ± 0.004	1.3 ± 0.4	0.86 ± 0.11	0.24 ± 0.02	0.19 ± <0.01
**ON-6**	0.066 ± 0.024	n.d. ^d^	0.021 ± 0.004	0.021 ± 0.001	0.011 ± 0.001
**ON-7**	0.022 ± 0.002	2.1 ± 0.1	0.83 ± 0.03	0.29 ± <0.01	0.18 ± <0.01
**ON-8**	0.058 ± 0.003	0.022 ± 0.012	0.078 ± 0.003	0.035 ± 0.002	0.024 ± <0.001

^a^ Conditions; 140 mM KCl, 10 mM MgCl_2_, 1.0 mM sodium phosphate, 10 mM sodium citrate-HCl buffer, 3.35 µM each strand, pH 4.0, 40°C; ^b^ Taken from the previous reports [28,29]; ^c^ **PDD** (parallel double-stranded DNA); 5’-d(GCTAAAAAGAAAGAGAGATCG)-3’/5’-d(CGATCTCTCTTTCTTTTTAGC)-3’; ^d^ Not determined due to low reactivity.

**Table 3:** Observed pseudo first-order rate constants of each oligonucleotide at pH 4.0, 20 °C ^a^.

ON	*k*_obs_ × 10^3^ (s^−1^) in the presence of		
	No template	PSD	ASR
**ON-1**	0.011 ± <0.001	0.27 ± 0.06	0.022 ± 0.001
**ON-2**	0.011 ± 0.002	0.0067 ± 0.0006	0.0059 ± 0.0006
**ON-3**	0.0072 ± 0.0010	0.19 ± 0.05	0.032 ± 0.002
**ON-4**	0.0062 ± 0.0011	n.d. ^b^	n.d. ^b^
**ON-5**	0.0073 ± 0.0015	0.26 ± 0.01	0.041 ± <0.001
**ON-6**	0.0067 ± 0.0013	n.d. ^b^	n.d. ^b^
**ON-7**	0.0080 ± 0.0008	0.68 ± 0.04	0.037 ± 0.005
**ON-8**	0.0070 ± 0.0005	n.d. ^b^	n.d. ^b^

^a^ Conditions; see Table 2, the reaction temperature was 20 °C.; ^b^ Not determined due to low reactivity.

#### 2.2.1. Reactivity on Parallel Single-Stranded DNA

The reactivity of **ON-1**–**ON-8** on **PSD** was similar to that on **PDD** (Table 2). The addition of 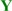 to the 5’-neighboring residue of 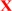 (the sequence 5’-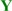

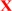
-3’ found in **ON-2** and **ON-4**) resulted in inactivation of hydrolysis upon hybridization to **PSD** (Figure S2). 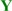 added to the 3’-neighboring residue of 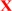 (5’-T
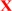

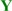
-3’) had little effect, resulting in equivalent *k*_obs_ for **ON-1** and **ON-3**. Accelerated hydrolysis of **ON-5** on **PSD** was observed as non-neighboring residual effects of 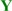. The sequence 5’-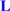

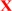
-3’ found in **ON-6** and **ON-8** eliminated the acceleration associated with hybridization to **PSD**, while hydrolysis of **ON-7** (sequence 5’-T
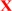

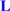
-3’) was accelerated (Figure 4). At 20 °C, **ON-1**–**ON-8** were less reactive, but the reactivity of **ON-7** was least affected, and reactivities of **ON-1**, **ON-3**, and **ON-5** were equivalent in the presence of **PSD** (Table 3, Figure S3).

#### 2.2.2. Reactivity on Anti-Parallel Single-Stranded DNA and RNA

In general, cleavage rate was lower in the presence of the anti-parallel single-stranded DNA or RNA than in the presence of **PDD** (Table 2). **ON-2**, **ON-4**, **ON-6**, and **ON-8** were not reactive in the presence of **ASD** or **ASR** (Figure S2). **ON-5** and **ON-7** were most reactive in the presence of **ASD**, but **ON-1**, **ON-3**, **ON-5**, and **ON-7** were all equally reactive in the presence of **ASR**. Although the reaction rate was slowed compared with that in the presence of **PDD**, **ON-7** showed 10 times higher reactivity in the presence of **ASD** than that in the absence of template (Figure 4). Lowering the reaction temperature to 20 °C resulted in significant decrease in reaction rate although the order of the reactivity was not affected (Table 3, Figure S3).

## 3. Discussion

Comparing the melting temperatures to the reactivities demonstrated that there seemed to be no direct correlation between the affinity to and reactivity on the templates. For examples, although **ON-3** always showed higher affinity and reactivity on the templates than **ON-2**, the more reactive **ON-7** exhibited lower affinity to the templates than non-reactive **ON-6** in the reaction with **PSD** and **ASR**. The absence of direct relationships between *T*_m_ at pH 6.0 and *k*_obs_ at pH 4.0 was consistent with the previous finding on **PDD** [29]. Although we expected that the extent of the strain on the P-N linkage might be reflected as a change in the thermal stability of the duplexes and triplexes, it was difficult to extract information on the change in microenvironment around a P-N linkage from the overall thermal stability of complex.

We observed a difference between the reaction with **PSD** and **ASR** in terms of response to the change of reaction temperature. Although the lower reaction temperature (20 °C) always slowed the rate of reaction, the extent of decrease was different between the reactions on **PSD** and **ASR** templates. In the reaction with **ASR**, the relative rates (ratio of *k*_obs_ at 20 °C to *k*_obs_ at 40 °C; *i.e.*, for the reaction with **ON-1** and **ASR**, *k*_obs_ was 0.022 and 0.17 at 20 °C and 40 °C, respectively, and the relative rate was 13%) varied from 13% to 22% for **ON-1**, **ON-3**, **ON-5**, and **ON-7**, and they varied from 30% to 82% in the reactions with **PSD**. This difference may indicate the presence of a substantial amount of unbound oligonucleotides in the reaction with **PSD** at 40 °C. However, judging from the melting experiments, we can estimate that the thermal stability of the duplexes with **PSD** was higher than that with **ASR** at pH 4.0, and the unbound fractions, if present, should be larger in the reactions with **ASR**. Moreover, the cleavage profiles at 40 °C seemed to follow pseudo first-order kinetics for both the reactions with **PSD** and **ASR**, indicating that substantial amounts of unbound oligonucleotides were not present (Figure S2). Thus, we suppose that the difference in the relative rates between the reactions with **PSD** and **ASR** was not due to thermal stability of the duplexes, but the temperature dependency of the reaction. Temperature dependency of these reactions may have depended on the activation energy of the cleavage and/or the temperature dependency of basicity of phosphoramidates.

The lability of the P-N linkage in duplexes was affected by both chemical modifications in oligonucleotides and the motif of the duplexes. Similar reactivities were observed in the reactions with **PSD** and those with **PDD**; this similarity indicated that the microenvironment around the P-N linkage resembled each other. This finding was not surprising because **PSD** was designed to form Hoogsteen-type parallel duplexes, which are a component of the reaction with **PDD**. Based on CD and IR spectra, the structure of triplexes and Hoogsteen duplexes are reportedly similar [38]. In contrast, **PSR** showed completely different behavior. While it is well known that triplexes and Hoogsteen duplexes are stabilized under acidic conditions [32,39,40,41], the stability of duplex **PSR**•**ON-0** was significantly lowered at pH 4.0 (Table 1, Figure S1). The absence of hysteresis for duplexes containing **PSR** was observed, but hysteresis was observed when duplex contained **PSD**. Moreover, triplexes containing RNA as a purine strand and DNA as a third strand did not form even in acidic conditions [42,43,44,45]. Therefore, we assume that **PSR** did not form Hoogsteen-type duplexes and that the duplexes with **PSR** were unstable under acidic conditions. Thus, **PSR** were apparently not suitable templates for acid-mediated hydrolysis of the phosphoramidate, and the reactivity with **PSR** templates was not evaluated.

**Figure 5:**
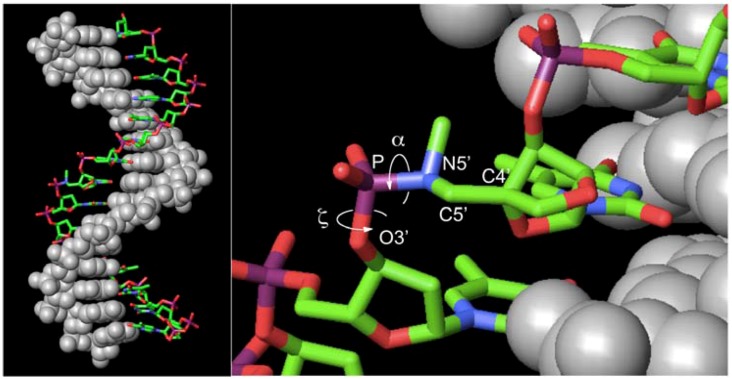
Molecular model of the **ON-1**•**ASD** duplex. **ON-1** and **ASD** rendered as stick (colored by element) and space-filling (gray) models, respectively. α (O3’-P-N5’-C5’) and ζ (C3’-O3’-P-N5’).

In the reactions with anti-parallel single-stranded DNA and RNA (**ASD** and **ASR**), the rate constants were as small as one-tenth of those in the reactions with **PDD**, indicating that the strain on the P-N linkage was not positioned appropriately. Recently, we proposed that the elevated basicity of the phosphoramidate may be responsible for accelerated hydrolysis [29]. From this point of view, the α and ζ dihedral angles would be important because they should have a significant impact on the electronic state of the phosphoramidate (Figure 5).

**Scheme 1:**
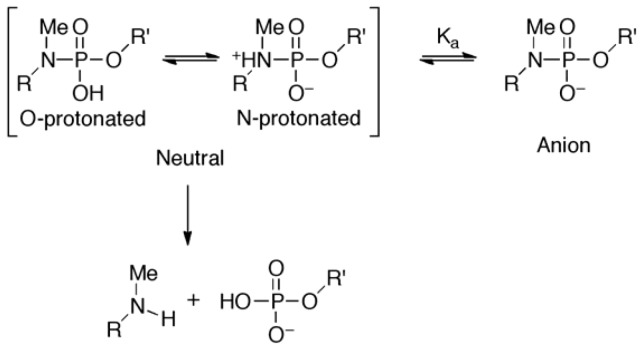
Equilibrium between neutral and anionic form and hydrolysis of phosphoramidate.

The duplexes containing **ASD** or **ASR** were likely to have B-form and A-form conformations [46], respectively, in which the α and ζ dihedral angles adopt –*sc* orientations [47]. The molecular models of **ON-1** with **ASD**, **ASR**, or **PDD** indicated that introduction of 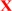 did not prohibit α and ζ dihedral angles from adopting −*sc* orientations (Figure 5, Figure S5). However, the high reactivity in triplexes and parallel Hoogsteen duplexes may have been due to a change in the preferred dihedral angles, which would make the phosphoramidates more basic. The quantum chemical calculations using one model compound (*N*,*N*,*O*-trimethylphosphoramidate, R = R’ = Me in Scheme 1) revealed the relative stability between the neutral form (N-protonated) and the anionic form of the phosphoramidate (Scheme 1) as functions of α and ζ. 

**Figure 6:**
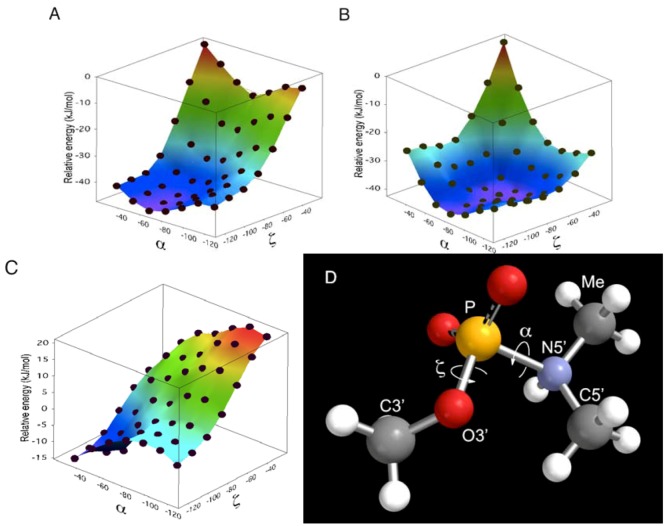
Relative energy of phosphoramidate model compound as function of α and ζ dihedral angles. The relative energy was calculated from the minimized structure at density functional B3LYP/6-311+G** level of theory. The calculation was performed *in vacuo* with the two dihedral angles corresponding to α and ζ constrained to angles ranging from −120° to −30° in increment of 15°. The relative energy profiles of the neutral form (**A**) and anionic form (**B**) are shown. The N-protonated (zwitterionic) form was used as the neutral form. The difference in relative energy between anionic form and neutral form (**C**) was calculated by subtraction of relative energy of anion form (**B**) from that of neutral form (**A**). The most basic conformer studied here is shown (**D**; α and ζ were –30° and –120°, respectively). α: O3’-P-N5’-C5’, ζ: C3’-O3’-P-N5’.

The N-protonated neutral form was considered to be the substrate in this reaction because the hydrolysis seemed to take place as a water molecule attacked from backside of the P-N linkage [28]. As is shown in Figure 6, the prevailing conformations in A- and B-form duplexes (α: −60°, ζ: −75° A-form/−90° B-form [47]) were calculated to be relatively stable in the anionic form. Increment and decrement in α and ζ dihedral angles, respectively, will favor the electrically neutral form (Figure 6, C). Especially, the basicity of the phosphoramidate was estimated to be strongest when the α and ζ dihedral angles were −30° and −120° (Figure 6D), respectively, and such a conformation might be preferred in the triplex and parallel Hoogsteen duplex, resulting in enhanced reactivity.

Although the structure of triplexes and parallel duplexes varies depending on the sequence and manner of determination [38,48,49], increased and decreased α and ζ dihedrals (α: −37° and ζ: −113°) are found in the model of parallel Hoogsteen duplex proposed by Sasisekharan *et al.* [38].

Our analysis of the effects of chemical modifications revealed that the orders of the reactivity of the oligonucleotides were nearly consistent regardless of the motifs of the duplexes. **ON-2**, **ON-4**, **ON-6,** and **ON-8** were always non-reactive, while **ON-5** and **ON-7** were the most reactive in the presence of templates. It seemed that the microenvironment around the P-N linkage was, to an extent, conserved in the right-handed helical structures studied here. Although the prediction of the precise conformation requires further investigation, the effects of chemical modifications could also be explained by alteration of conformational preference. For example, 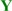 is known to affect the sugar conformation of 3’-adjacent nucleotide inducing C3’-*endo* conformation [50,51]. Although such an effect is not the case in **ON-2** and **ON-4** because 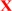 is pre-locked to C3’-*endo* conformation due to the 2’,4’-bridge moiety [26], 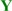 at 5’-adjacent of the phosphoramidate will have structurally affected the preferred conformation of the phosphoramidate, which resulted in inactivation of **ON-2** and **ON-4** in the presence of templates.

## 4. Experimental Section

### 4.1. Preparation of Oligonucleotides

Natural oligonucleotides and those with 2’-deoxy-5-methylcytidine (5-MedC), such as the template DNA (**PSD**, **ASD**), RNA (**PSR**, **ASR**), and **ON-0**, were purchased from Hokkaido System Science Co., Ltd., Sapporo, Japan. Oligonucleotides containing 5’-amino-2’,4’-BNA (**ON-1**–**ON-8**) were synthesized as described previously [29]. 

### 2.2. UV Melting Experiments

UV melting experiments were performed using Shimadzu UV-1650 and Shimadzu UV-1800 spectrometers. Oligonucleotides were dissolved in a solution buffered to pH 6.0 or 4.0 containing 140 mM KCl, 10 mM MgCl_2_, 1.0 mM sodium phosphate, and 10 mM sodium citrate (pH 6.0) or 10 mM sodium citrate-HCl (pH 4.0). The final concentration of each oligonucleotide was 1.5 µM. Solutions containing the oligonucleotides were heated and subsequently cooled to 10 °C to generate duplexes. Each solution was heated and subsequently cooled from 10 °C to 90 °C to 10 °C at the rate of 0.5 °C/min, and the hyperchromic changes were monitored at 260 nm. The melting temperatures (*T*_m_s) were determined as the intersection of the melting curve and the median of lower and higher base lines derived from the heating processes (Table 1, Figure 3, Figure S1).

### 4.3. Hydrolysis Experiments

Hydrolysis experiments were performed as described previously [28,29]. The reaction solutions contained 140 mM KCl, 10 mM MgCl_2_, 1.0 mM sodium phosphate, 10 mM sodium citrate-HCl, and 3.35 µM of each strand. The final pH was 4.0. Reaction solutions were kept at 40 °C or 20 °C for 0, 30, 60, 120, and 180 min, and each reaction solution (10 µL) was diluted with 100 mM glycine-NaOH (pH 9.0, 160 µL). The diluted samples were analyzed by reversed-phase HPLC (Shimadzu Prominence LC-20 system, Kyoto, Japan) to determine the percent of intact oligonucleotides (Table 2, Table 3, Figure 4, Figure S2, S3, S4).

### 4.4. Molecular Modeling and Computation

The initial structures for molecular modeling, **ON-1**•**ASD**, **ON-1**•**ASR**, and **ON-1**•**PDD**, were generated with Discovery Studio 3.1^TM^ (Accelrys Software, Inc., San Diego, CA, USA) using default parameters for B-form DNA•DNA duplexes, A-form DNA•RNA duplexes, and triplexes, respectively. In the initial models, **ON-1** contained 2’,4’-BNA/LNA in place of 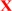. The structures generated were exported to MacroModel 9.1^TM^ (Schrödinger, LLC, New York, NY, USA). An energy minimization calculation was performed for each structure using 1) AMBER* as a force field, 2) the GB/SA solvation model of water, and 3) the PRCG method to obtain structures optimized to within a gradient of 0.05 kJ/molÅ. Finally, the 5’-oxygen of 2’,4’-BNA/LNA in **ON-1** of the optimized structures was replaced by nitrogen attached to a methyl group to obtain the molecular models (Figure 5, Figure S5).

The quantum mechanical calculations were performed with Spartan’08 for Mac (Wavefunction, Inc., Irvine, CA, USA). The two dihedral angles of *N*,*N*,*O*-trimethylphosphoramidate corresponding to α and ζ were constrained to angles ranging from −120° to −30° in increments of 15°. The constrained structures were subject to geometric optimization at semi-empirical PM3, HF/6-31+G* and density functional B3LYP/6-311+G** level of theory. All calculations were performed *in vacuo* (Figure 6).

## 5. Conclusions

We demonstrated that duplex formation induced conformational strain on the P-N linkage in oligonucleotides. Parallel Hoogsteen duplex formation brought higher reactivity than Watson-Crick duplex formation. The introduction of 2’,5’-linked DNA at the 3’-neighboring residue of 5’-amino-2’,4’-BNA promoted high reactivity in the presence of parallel and anti-parallel single-stranded DNA, as well as with parallel dsDNA templates.

## Supplementary Materials

Supplementary materials can be accessed at: http://www.mdpi.com/1420-3049/16/12/10695/s1.

## References

[1] Xu Y., Karalkar N.B., Kool E.T. (2001). Nonenzymatic autoligation in direct three-color detection of RNA and DNA point mutations. Nat. Biotechnol..

[2] Sando S., Kool E.T. (2002). Quencher as leaving group: Efficient detection of DNA-joining reactions. J. Am. Chem. Soc..

[3] Abe H., Kool E.T. (2004). Destabilizing universal linkers for signal amplification in self-ligating probes for RNA. J. Am. Chem. Soc..

[4] Ficht S., Dose C., Seitz O. (2005). As fast and selective as enzymatic ligations: Unpaired nucleobases increase the selectivity of DNA-controlled native chemical PNA ligation. ChemBioChem.

[5] Dose C., Ficht S., Seitz O. (2006). Reducing product inhibition in DNA-template-controlled ligation reactions. Angew. Chem. Int. Ed..

[6] Abe H., Kool E.T. (2006). Flow cytometric detection of specific RNAs in native human cells with quenched autoligating FRET probes. Proc. Natl. Acad. Sci. USA.

[7] Ogasawara S., Fujimoto K. (2006). SNP genotyping by using photochemical ligation. Angew. Chem. Int. Ed..

[8] Yoshimura Y., Noguchi Y., Sato H., Fujimoto K. (2006). Template-directed DNA photoligation in rapid and selective detection of RNA point mutations. ChemBioChem.

[9] Peng X., Greenberg M.M. (2008). Facile SNP detection using bifunctional, cross-linking oligonucleotide probes. Nucleic Acids Res..

[10] Abe H., Kondo Y., Jinmei H., Abe N., Furukawa K., Uchiyama A., Tsuneda S., Aikawa K., Matsumoto I., Ito Y. (2008). Rapid DNA chemical ligation for amplification of RNA and DNA signal. Bioconjug. Chem..

[11] Dose C., Seitz O. (2008). Single nucleotide specific detection of DNA by native chemical ligation of fluorescence labeled PNA-probes. Bioorg. Med. Chem..

[12] Grossmann T.N., Seitz O. (2006). DNA-catalyzed transfer of a reporter group. J. Am. Chem. Soc..

[13] Grossmann T.N., Röglin L., Seitz O. (2008). Angew. Chem. Int. Ed..

[14] Grossmann T.N., Seitz O. (2009). Nucleic acid templated reactions: Consequences of probe reactivity and readout strategy for amplified signaling and sequence selectivity. Chem. Eur. J..

[15] Brunner J., Mokhir A., Kraemer R. (2003). DNA-templated metal catalysis. J. Am. Chem. Soc..

[16] Boll I., Krämer R., Brunner J., Mokhir A. (2005). Templated metal catalysis for single nucleotide specific DNA sequence detection. J. Am. Chem. Soc..

[17] Franzini R.M., Kool E.T. (2009). Efficient nucleic acid detection by templated reductive quencher release. J. Am. Chem. Soc..

[18] Cai J., Li X., Yue X., Taylor J.S. (2004). Nucleic acid-triggered fluorescent probe activation by the Staudinger reaction. J. Am. Chem. Soc..

[19] Cai J., Li X., Taylor J.S. (2005). Improved nucleic acid triggered probe activation through the use of a 5-thiomethyluracil peptide nucleic acid building block. Org. Lett..

[20] Pianowski Z.L., Winssinger N. (2007). Fluorescence-based detection of single nucleotide permutation in DNA via catalytically templated reaction. Chem. Commun..

[21] Franzini R.M., Kool E.T. (2008). 7-Azidomethoxy-coumarins as profluorophores for templated nucleic acid detection. ChemBioChem.

[22] Franzini R.M., Kool E.T. (2008). Organometallic activation of a fluorogen for templated nucleic acid detection. Org. Lett..

[23] Furukawa K., Abe H., Wang J., Uda M., Koshino H., Tsuneda S., Ito Y. (2009). Reduction-triggered red fluorescent probes for dual-color detection of oligonucleotide sequences. Org. Biomol. Chem..

[24] Prusty D.K., Herrmann A. (2010). A fluorogenic reaction based on heavy-atom removal for ultrasensitive DNA detection. J. Am. Chem. Soc..

[25] Li X., Liu D.R. (2004). DNA-templated organic synthesis: Nature’s strategy for controlling chemical reactivity applied to synthetic molecules. Angew. Chem. Int. Ed..

[26] Obika S., Nakagawa O., Hiroto A., Hari Y., Imanishi T. (2003). Synthesis and properties of a novel bridged nucleic acid with a P3’→N5’ phosphoramidate linkage, 5’-amino-2’,4’-BNA. Chem. Commun..

[27] Obika S., Tomizu M., Negoro Y., Osakai T., Orita A., Ueyama Y., Nakagawa O., Imanishi T. (2007). Acid-mediated cleavage of oligonucleotide P3’→N5’ phosphoramidates triggered by sequence-specific triplex formation. Nucleos. Nucleot. Nucleic Acids.

[28] Obika S., Tomizu M., Negoro Y., Orita A., Nakagawa O., Imanishi T. (2007). Double-stranded DNA-templated digestion triggered by triplex formation. ChemBioChem.

[29] Ito K.R., Kodama T., Tomizu M., Negoro Y., Orita A., Osaki T., Hosoki N., Tanaka T., Imanishi T., Obika S. (2010). Double-stranded DNA-templated cleavage of oligonucleotides containing a P3’→N5’ linkage triggered by triplex formation: The effects of chemical modifications and remarkable enhancement in reactivity. Nucleic Acids Res..

[30] Obika S., Nanbu D., Hari Y., Andoh J., Morio K., Doi T., Imanishi T. (1998). Stability and structural features of the duplexes containing nucleoside analogues with a fixed N-type conformation, 2’-*O*,4’-*C*-methyleneribonucleosides. Tetrahedron Lett..

[31] Singh S.K., Nielsen P., Koshkin A.A., Wengel J. (1998). LNA (locked nucleic acids): Synthesis and high-affinity nucleic acid recognition. Chem. Commun..

[32] Bhattacharyya J., Maiti S., Muhuri S., Nakano S., Miyoshi D., Sugimoto N. (2011). Effect of locked nucleic acid modifications on the thermal stability of noncanonical DNA structure. Biochemistry.

[33] Rizzo C.J., Dougherty J.P., Breslow R. (1992). 3’-Deoxy-2’-phosphoramidites of adenosine and 5-methyluridine used for the solid phase synthesis of unnatural 3’-deoxy-2’-5’’-oligonucleotides. Tetrahedron Lett..

[34] Dougherty J.P., Rizzo C.J., Breslow R. (1992). Oligodeoxynucleotides that contain 2’,5’’ linkages: Synthesis and hybridization properties. J. Am. Chem. Soc..

[35] Giannaris P.A., Damha M.J. (1993). Oligoribonucleotides containing 2’,5’-phosphodiester linkages exhibit binding selectivity for 3’,5’-RNA over 3’,5’-ssDNA. Nucleic Acids Res..

[36] Prakash T.P., Jung K., Switzer C. (1996). RNA recognition by the 2’-structural isomer of DNA. Chem. Commun..

[37] Sheppard T.L., Breslow R.C. (1996). Selective binding of RNA, not DNA, by complementary 2’,5’-linked DNA. J. Am. Chem. Soc..

[38] Raghunathan G., Miles H.T., Sasisekharan V. (1994). Parallel nucleic acid helices with Hoogsteen base pairing: Symmetry and structure. Biopolymers.

[39] Singleton S.F., Dervan P.B. (1992). Influence of pH on the equilibrium association constants for oligodeoxyribonucleotide-directed triple helix formation at single DNAsites. Biochemistry.

[40] Hashem G.M., Wen J., Do Q., Gray D.M. (1999). Evidence from CD spectra and melting temperatures for stable Hoogsteen-paired oligomer duplexes derived from DNA and hybrid triplexes. Nucleic Acids Res..

[41] Sugimoto N., Wu P., Hara H., Kawamoto Y. (2001). pH and cation effects on the properties of parallel pyrimidine motif DNA triplexes. Biochemistry.

[42] Roberts R.W., Crothers D.M. (1992). Stability and properties of double and triple helices: Dramatic effects of RNA or DNA backbone composition. Science.

[43] Han H., Dervan P.B. (1993). Sequence-specific recognition of double helical RNA and RNA•DNA by triple helix formation. Proc. Natl. Acad. Sci. USA.

[44] Escudé C., François J., Sun J., Ott G., Sprinzl M., Garestier T., Hélène C. (1993). Stability of triple helices containing RNA and DNA strands: experimental and molecular modeling studies. Nucleic Acids Res..

[45] Han H., Dervan P.B. (1994). Different conformational families of pyrimidine•purine•pyrimidine triple helices depending on backbone composition. Nucleic Acids Res..

[46] Xiong Y., Sundaralingam M. (1998). Crystal structure and conformation of a DNA-RNA hybrid duplex with a polypurine RNA strand: d(TTCTTBr^5^CTTC)-r(GAAGAAGAA). Structure.

[47] MacKerell A.D. (2009). Contribution of the intrinsic mechanical energy of the phosphodiester linkage to the relative stability of the A, B_I_, and B_II_ forms of duplex DNA. J. Phys. Chem. B.

[48] Bhaumik S.R., Chary K.V.R., Govil G., Liu K., Miles H.T. (1998). A novel palindromic triple-stranded structure fromed by homopyrimidine dodecamer d-CTTCTCCTCTTC and homopurine hexamer d-GAAGAG. Nucleic Acids Res..

[49] Rhee S., Han Z., Liu K., Miles H.T., Davies D.R. (1999). Structure of a triple helical DNA with a triplex-duplex junction. Biochemistry.

[50] Petersen M., Nielsen C.B., Nielsen K.E., Jensen G.A., Bondensgaard K., Singh S.K., Rajwanshi V.K., Koshkin A.A., Dahl B.M., Wengel J. (2000). The conformations of locked nucleic acids (LNA). J. Mol. Recognit..

[51] Jensen G.A., Singh S.K., Kumar R., Wengel J., Jacobsen J.P. (2001). A comparison of the solution structures of an LNA:DNA duplex and the unmodified DNA:DNA duplex. J. Chem. Soc. Perkin Trans. 2.

