# Supplementary material for: Cleavage of Oligonucleotides Containing a P3’→N5’ Phosphoramidate Linkage Mediated by Single-Stranded Oligonucleotide Templates
Source: Molecules. 2011 Dec 20;16(12):10695–708. doi: 10.3390/molecules161210695 (PMC6264227; doi:10.3390/molecules161210695)
Supplement: Supplementary file 1 [file molecules-16-10695-s001.pdf]

Supplementary Materials

1. UV melting experiments

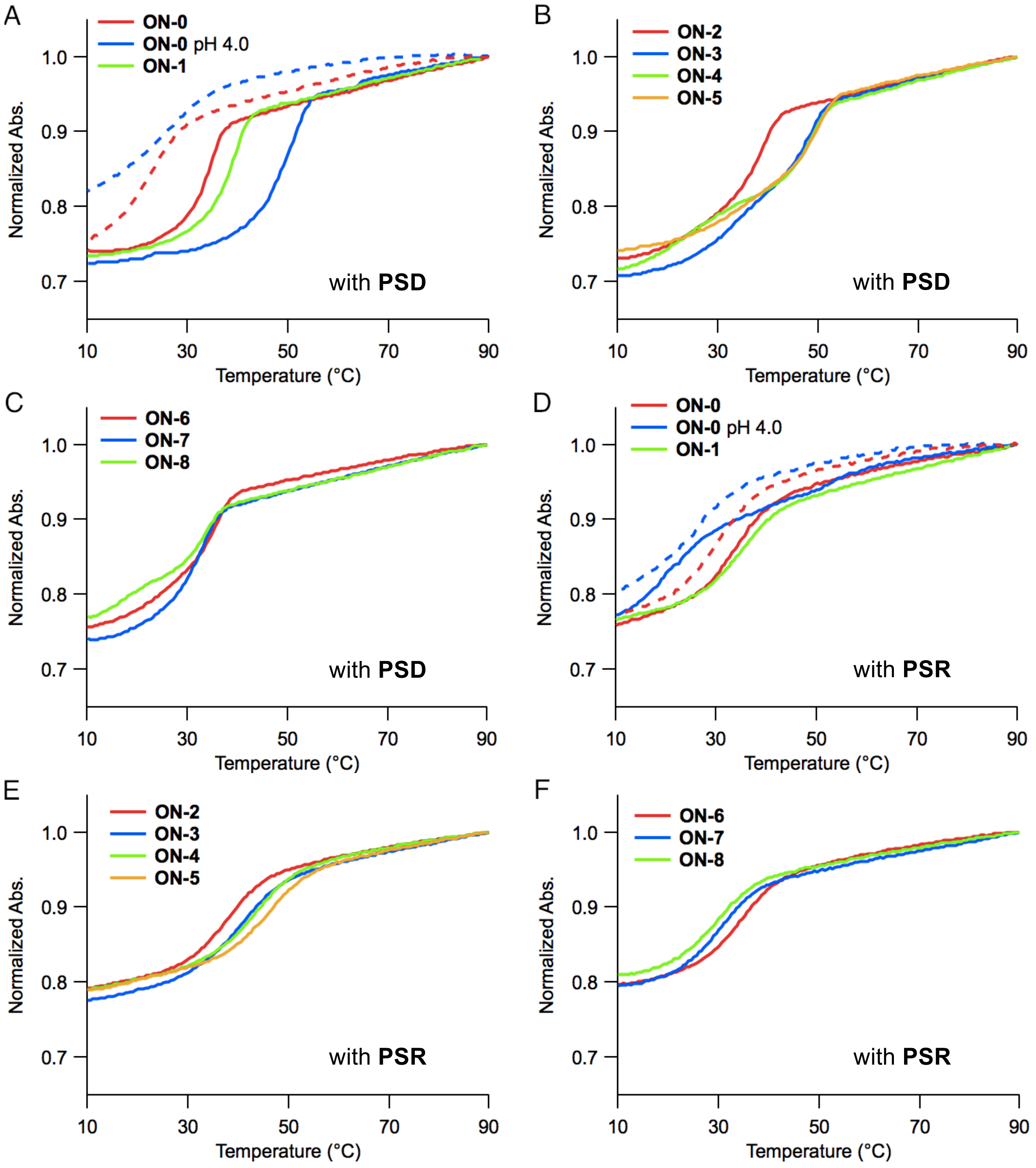

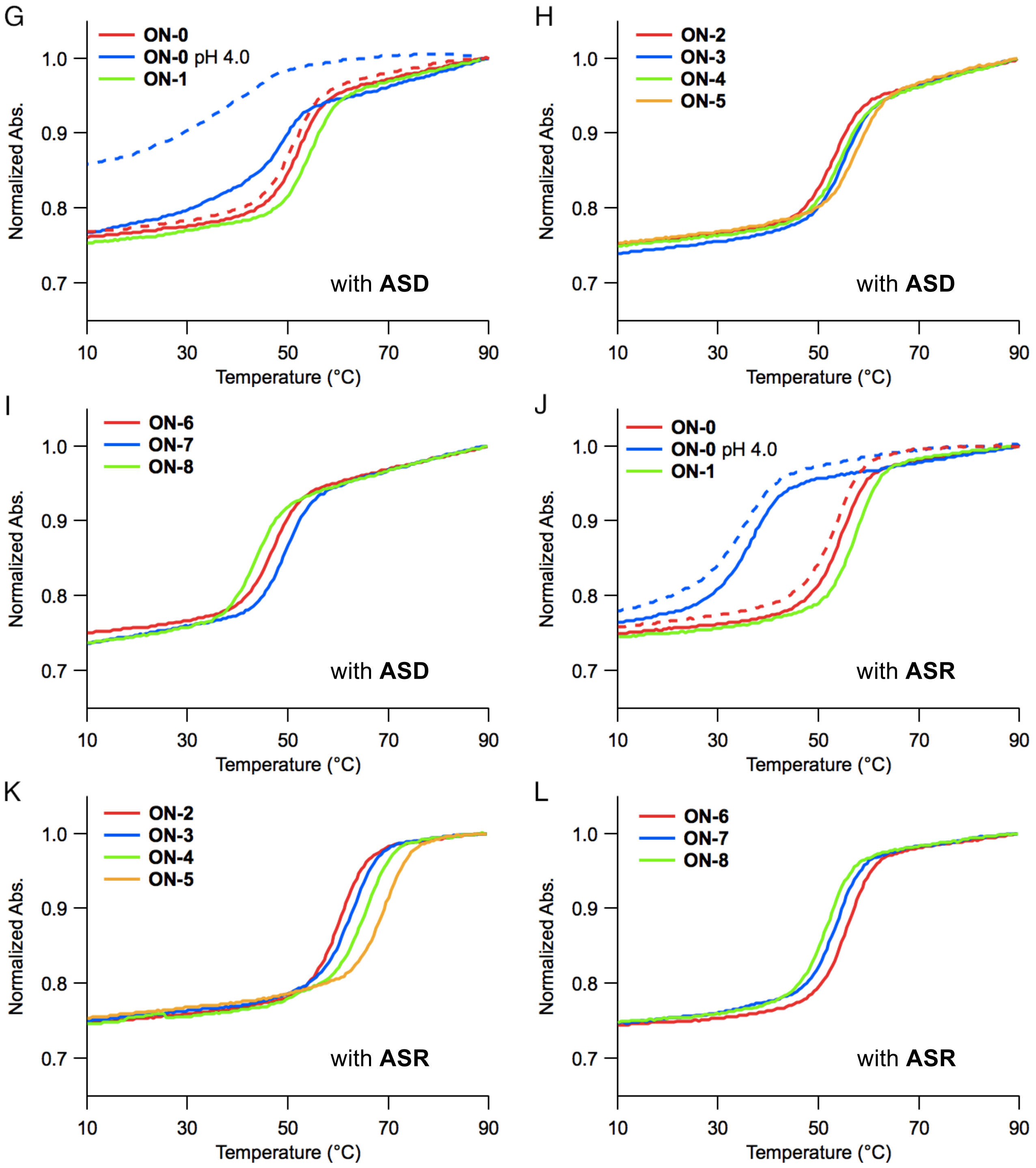

Figure S1. Melting profiles of **ON-0**–**ON-8** with **PSD** (A–C), **PSR** (D–F), **ASD** (G–I) and **ASR** (J–L). Melting experiments were performed in a solution buffered to pH 6.0 unless otherwise specified. The cooling processes are given for the duplexes containing **ON-0** by dashed lines. Conditions: 1.5 µM each strand, 140 mM KCl, 10 mM $MgCl_2$, 1.0 mM sodium phosphate and 10 mM sodium citrate (pH 6.0) or 10 mM sodium citrate-HCl (pH 4.0).

2. Hydrolysis experiments

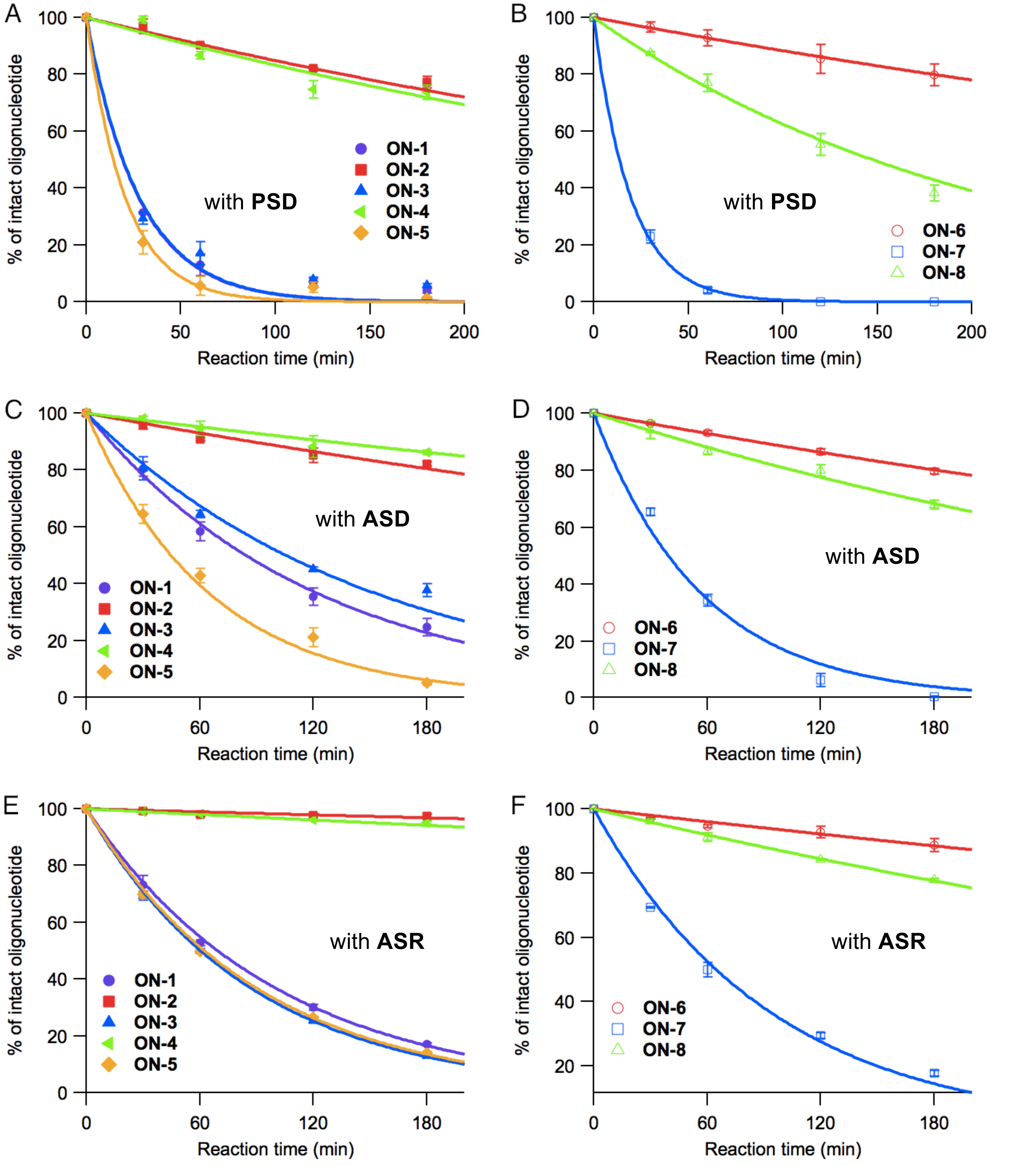

Figure S2. Cleavage profiles of **ON-1**–**ON-8** in the presence of **PSD** (A, B), **ASD** (C, D) and **ASR** (E, F) at pH 4.0, 40°C.

Hydrolysis experiments were performed in a solution buffered to pH 4.0 containing 140 mM KCl, 10 mM $MgCl_2$, 1.0 mM sodium phosphate, 10 mM sodium citrate-HCl, 3.35 μM each strand, at 40°C.

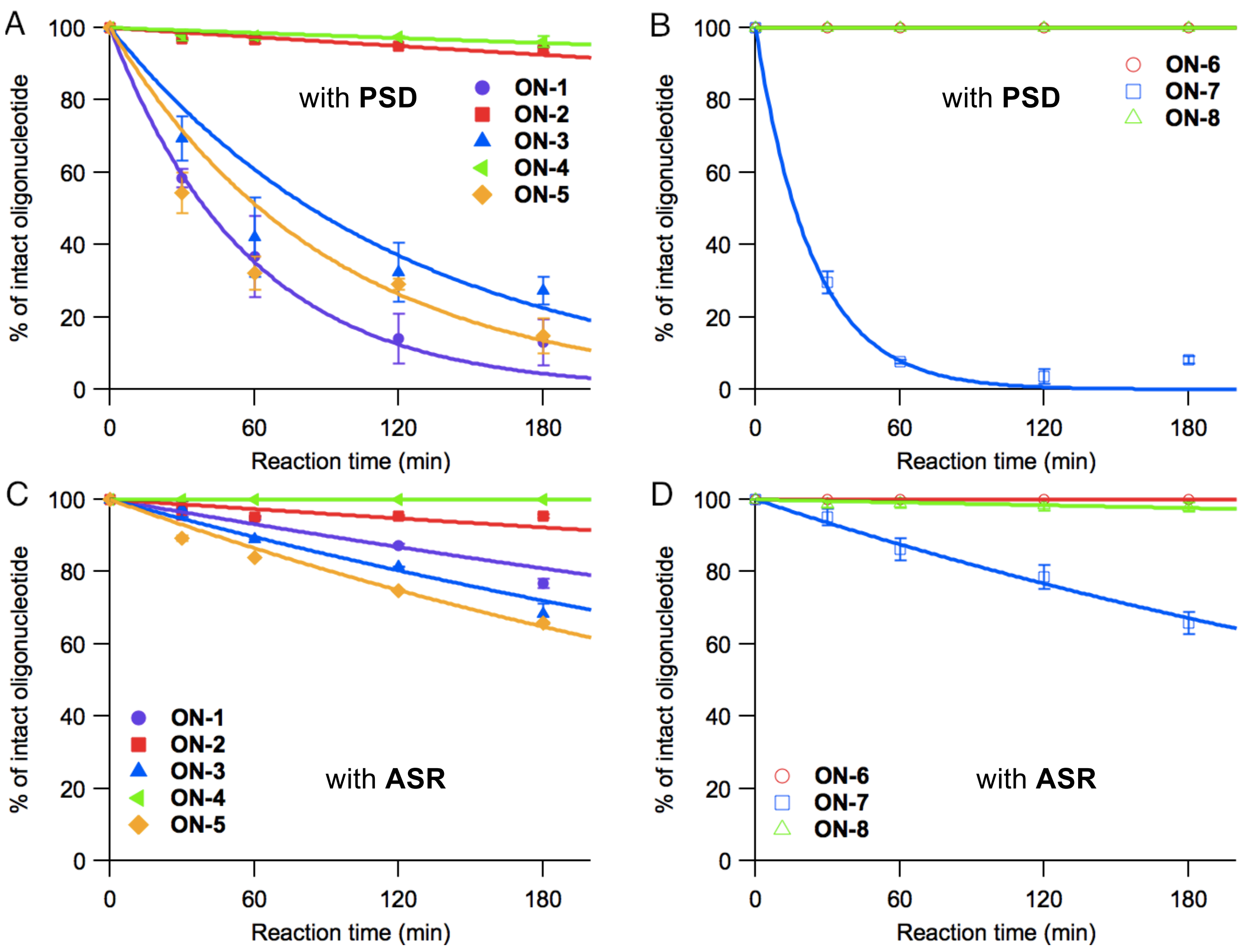

Figure S3. Cleavage profiles of **ON-1**–**ON-8** in the presence of **PSD** (A, B) and **ASR** (E, F) at pH 4.0, 20°C.
Hydrolysis experiments were performed at 20°C, see Figure S2 caption.

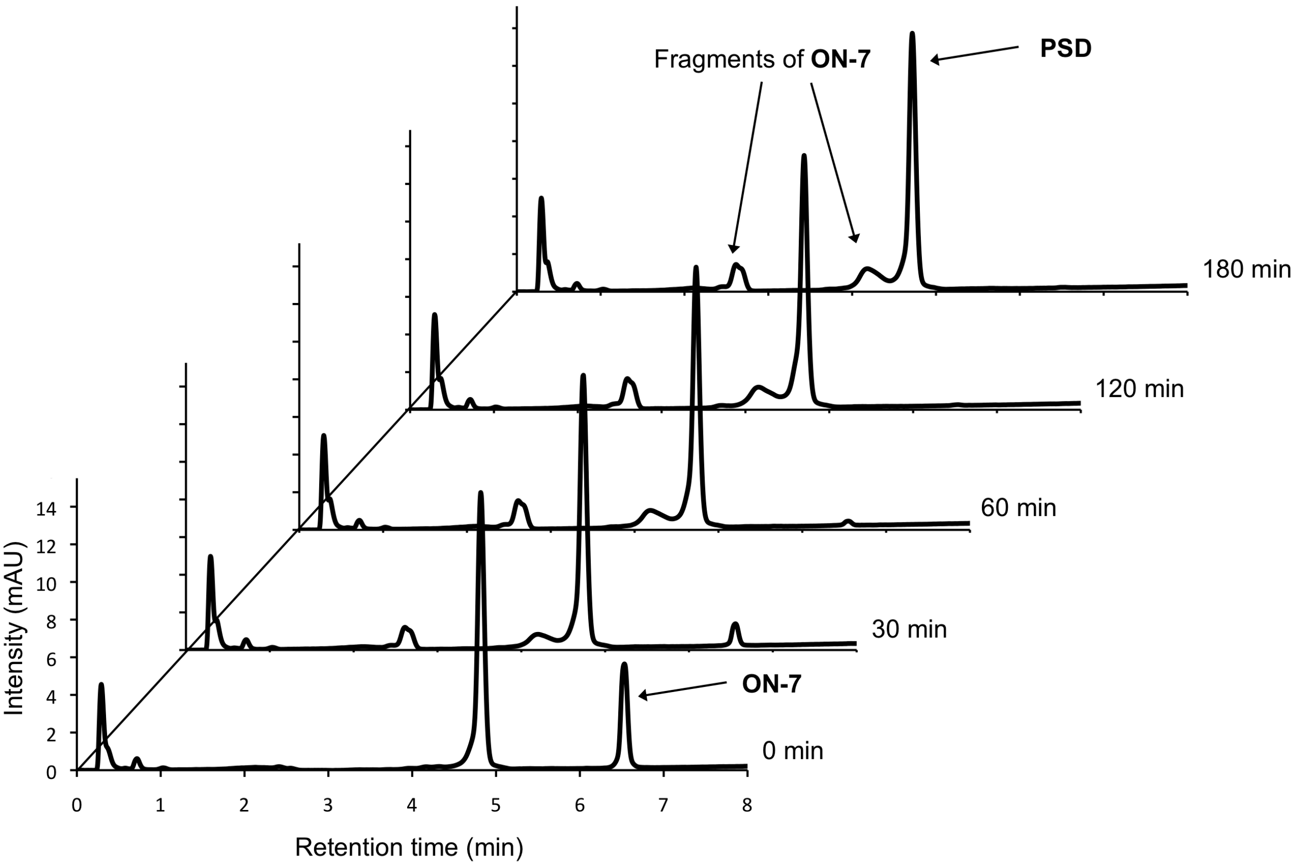

Figure S4. HPLC profile of the reaction of **ON-7** with **PSD** at 40°C.

The HPLC system used for the analysis of the reactions was composed of the following components: a Shimadzu CTO-20A column oven, a SPD-20A UV detector, a LC-20AB pump, a SIL-20A auto sampler, a DGU-20A3 online degasser, CBM-20A system controller and an LCsolution HPLC workstation software. Reversed-phase HPLC was run using a Kinetex^{TM} C18 column (2.6 µm 3.0×50 mm) [buffer **A**, 0.1 M TEAA (pH 7.0); buffer **B**, 0.1 M TEAA (pH 7.0)/MeCN = 1:1; linear gradient, **B** 14 to 30%/7 min; flow rate, 1.0 mL/min; detection, 260 nm].

3. Molecular modeling

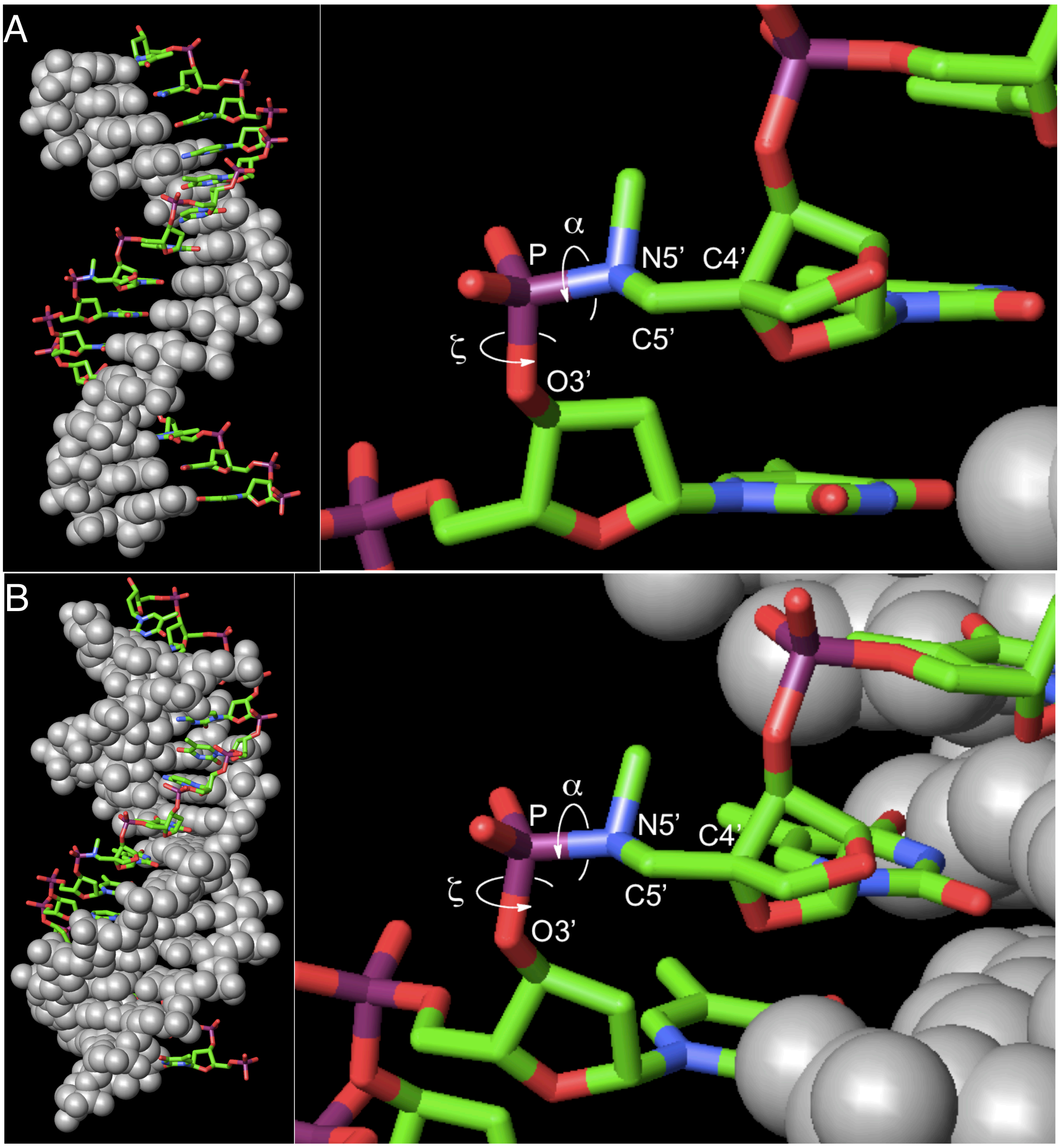

Figure S5. Molecular models of the duplex **ON-1•ASR** (A) and the triplex **ON-1•PDD** (B). **ON-1** rendered as stick models (colored by element), **ASR** and **PDD** rendered as space-filling models (gray). α: O3'-P-N5'-C5', ζ: C3'-O3'-P-N5'.
